# Cytokine Gene Polymorphisms across Tuberculosis Clinical Spectrum in Pakistani Patients

**DOI:** 10.1371/journal.pone.0004778

**Published:** 2009-03-10

**Authors:** Ambreen Ansari, Najeeha Talat, Bushra Jamil, Zahra Hasan, Tashmeem Razzaki, Ghaffar Dawood, Rabia Hussain

**Affiliations:** 1 Pathology & Microbiology Department, The Aga Khan University, Karachi, Pakistan; 2 Pathology, Microbiology & Medicine Department, The Aga Khan University, Karachi, Pakistan; 3 Sindh Institute of Urology and Transplantation, Karachi, Pakistan; 4 Masoomeen General Hospital, Karachi, Pakistan; Columbia University, United States of America

## Abstract

**Background:**

Pakistan ranks 7^th^ globally in terms of tuberculosis (TB) disease burden (incidence 181/100000 pop./yr; prevalence of 329/pop./yr). Reports from different populations show variable associations of TB susceptibility and severity with cytokine gene polymorphisms. Tuberculosis clinical severity is multi-factorial and cytokines play a pivotal role in the modulation of disease severity. We have recently reported that the ratio of two key cytokines (IFNγ and IL10) show significant correlation with the severity spectrum of tuberculosis. The objective of the current study was to analyze the frequency of cytokine gene polymorphisms linked to high and low responder phenotypes (IFNγ +874 *T* ^hi^→*A* ^lo^ and IL10 −1082 *G* ^lo^→*A* ^hi^) in tuberculosis patients.

**Methods and Findings:**

Study groups were stratified according to disease site as well as disease severity: Pulmonary N = 111 (Minimal, PMN = 19; Moderate, PMD = 63; Advance, PAD = 29); Extra-pulmonary N = 67 (Disseminated DTB = 20, Localized LTB = 47) and compared with healthy controls (TBNA = 188). Genotype analyses were carried out using amplification refractory mutation system-PCR (ARMS-PCR) and stimulated whole blood (WB) culture assay was used for assessing cytokine profiles. Our results suggest that the IFNγ +874 *TT* genotype and *T* allele was overrepresented in PMN (p = 0.01) and PMD (p = 0.02). IFNγ +874 *TT* in combination with IL10 *GG* ^lo^ genotypes showed the highest association (χ^2^ = 6.66, OR = 6.06, 95% CI = 1.31–28.07, *p* = 0.01). IFNγ *AA* ^lo^ on the other hand in combination with IL10 *GG* ^lo^ increased the risk of PAD (OR = 5.26; p = 0.005) and DTB (OR = 3.59; p = 0.045).

**Conclusion:**

These findings are consistent with the role of IL10 in reducing collateral tissue damage and the protective role of IFNγ in limiting disease in the lung.

## Introduction

Pakistan ranks 7^th^ globally in terms of tuberculosis disease burden with an incidence of 181/100000 pop./yr and a prevalence of 329/ pop. /yr. Several reports from different countries have shown that household contacts of active pulmonary tuberculosis are at much higher risk of latent infection that range from 30–80% depending on the intensity of tuberculosis disease transmission [1]–[4]. Only ten percent of those latently infected individuals develop TB disease during their lifetime [5]. Identification of these high-risk individuals in recently exposed/ infected individuals is of great importance to TB Control Programs for reducing the disease burden in the community. Association of pathological severity with increased circulating levels of different pro-inflammatory and or down-regulatory cytokines in tuberculosis is fairly well established [6]. An increasing number of studies have shown that single nucleotide polymorphisms (SNPs) located in the promoter or coding regions of cytokine genes result in differential cytokine secretion due to altered transcriptional activation. In humans, families with SNPs in IFNγ receptor 1 [7], [8] genes have shown Mendelian susceptibility to tuberculosis. Single nucleotide polymorphisms (SNPs) located in the first intron of the IFNγ gene (at position +874) have shown variable associations with tuberculosis disease susceptibility and severity [9]. IFNγ (+874*T* ^hi^→*A* ^lo^) polymorphism is located within a putative NF-kb binding site and shows preferential binding to the *T* allele and correlates with high IFNγ producer phenotype [10]. Similarly polymorphisms in IL10 [11] linked to high and low producer phenotypes have shown conflicting associations with tuberculosis disease susceptibility and disease severity in different patient populations [12], [13]. This is not surprising as, although IFNγ may be a key cytokine in activation of macrophages for mycobacterial stasis and killing [14], disease severity outcomes in tuberculosis depend on the balance among several different cytokines *in situ*, depending on the disease site. IL10 is particularly important in reducing collateral tissue damage, particularly in the lung by dampening macrophage activation and by indirectly antagonizing IFNγ function [15]. We have recently reported that the ratio of these two key cytokines (IFNγ/IL10) shows significant correlation with clinical severity in extra-pulmonary tuberculosis [16],with higher IFNγ/IL10 ratio relating to less severe disease. We have now extended these studies to analyze the frequency of high and low responder cytokine phenotypes (IFNγ +874 *T* ^hi^→*A* ^lo^ and IL10 −1082 *G* ^lo^→*A* ^hi^) to analyze the relationship of these SNPs with clinical severity of tuberculosis. Our results show that SNPs in IFNγ are significantly related to site of TB disease (Pulmonary *vs.* Extrapulmonary) while combinations of SNPs in IFNγ and IL10 genes are important determinants of TB disease severity. Our results therefore substantiate our aim of the study.

## Results

### Demographic characteristics of the study groups

There were no significant differences in age in TBNA and TBA groups (Table 1). The ratio of females was significantly higher (*p*<0.05) in TBA compared to TBNA. However, no significant association of genotype with age or sex was observed in both TBA and TBNA (>0.05) using multiple logistic regression analysis (data not shown).

**Table 1:** Demographic characteristics of TB Patients and Controls.

Group Studied	N	Gender		Age (years)	
		Female (%)	Male (%)	Mean	Range
TB not affected controls (TBNA)	188	88 (46.8)	100 (53.1)	28.10	6–70
TB affected patients (TBA)	188	118 (62.7)	70 (37.2)	33.40	7–81
**Pulmonary TB (PTB)**	111	65 (58.5)	46 (41.4)	32.0	10–81
Pulmonary minimal (PMN)	19	9 (47.3)	10 (52.6)	36.3	15–81
Pulmonary moderate (PMD)	63	37 (58.7)	26 (41.2)	33.0	13–70
Pulmonary advance (PAD)	29	19 (65.5)	10 (34.4)	26.2	10–69
**Extra pulmonary TB (ETB)**	67	47 (70.1)	20 (35.0)	34.5	7–80
Disseminated TB (DTB)	20	12 (60.0)	8 (40.0)	37.3	16–80
Localized TB (LTB)	47	35 (74.5)	12 (25.5)	33.3	7–75

**Note:** Patient stratification is as given in Materials and Methods. Abbreviations for groups are given in brackets. TBA included 10 previously treated patients.

### Relationship of genotype and phenotype of IFNγ (+874*T* ^hi^→*A* ^lo^) and IL10 (−1082 *G* ^lo^→*A* ^hi^) in TBNA

**Figure 1:**
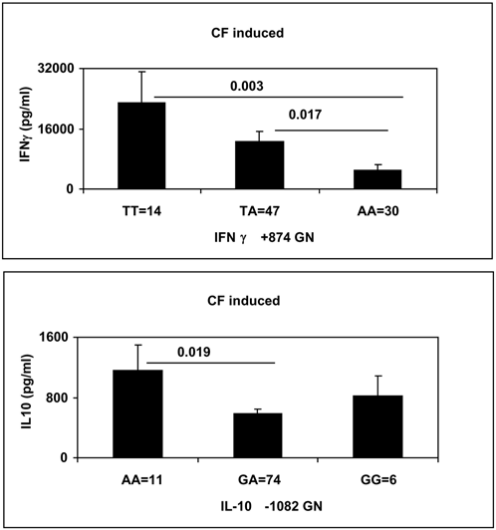
Relationship of IFNγ and IL10 SNPs with mycobacterial antigen induced cytokine secretion. Whole blood from TB not affected (TBNA) Tuberculin skin test positive donors was stimulated with *M. tuberculosis* culture filtrate (CF) proteins [5 µg/ml] and supernatants tested at 2 days for IL10 secretion and day 5 for IFNγ secretion using ELISA method as described in material and methods. Results are expressed as pg/ml after deducting secretion in un-stimulated whole blood.

Figure 1 shows the relationship of cytokine secretion in response to mycobacterial antigen stimulation with different genotypes IFNγ (+874 *T^hi^*→*A* ^lo^) and IL10 (−1082 *G* ^lo^→*A* ^hi^).We found IFNγ *TT* and IL10 *AA* to be the high producer phenotype in response to mycobacterial antigens. We next looked at the frequency of these genotypes in association with disease severity.

### Distinct IFNγ +874 but not IL10 −1082 genotype SNPs are associated with different disease sites and not with TB susceptibility

Cytokine genotypes and alleles frequencies in all patient groups were compared with healthy controls (Tables 3 & 4). IFNγ +874*T*→*A* genotype distribution was in Hardy-Weinberg equilibrium for both patients and controls (TBA; *p* = 0.607 and TBNA; *p* = 1.0). On the other hand, IL-10 −1082G→A polymorphism showed deviation from HWE due to excess of heterozygosity (TBA and TBNA; *p* = 0.00004). Cases and controls were compared using χ^2^, odds ratios (OR) and their confidence intervals, (95% CI). A *p* value of <0.05 was considered significant. Different cytokine genotypes and alleles showed distinct associations with different disease types as described below.

IFNγ +874 *TT* genotype showed significant association with PTB (χ^2^ = 6.05, *p* = 0.034) (Table 3). This association was restricted to the T allele (Table 4). In terms of disease severity in the lung this association was restricted to PMN and PMD and was not seen with PAD. Similarly, no association was observed with ETB or stratified ETB on the basis of disease severity (DTB *vs.* LTB). When we compared healthy controls with latent infection (TST+) similar and much more marked effect of IFNγ +874 *TT* genotype was observed (Table 3). Therefore, genotype associations were not only different according to disease site (PTB *vs.* ETB) but there was a distinct difference between severity within a disease site (PMD *vs.* PAD). These results therefore suggest that it is inappropriate to pool tuberculosis patient with differing sites and even severity within individual sites.

Table 3 also shows the frequency of IL10 −1082 genotype in relation to disease site as well as disease severity. Neither IL10 −1082 genotype nor alleles (Table 4) show any association in relation to either site or severity of tuberculosis. The *p* values were similar when patients were compared with TST+ controls (Table 4). There was an overrepresentation of the IL10 −1082 heterozygous genotype *GA* in both controls and PTB as well as ETB. Linkage disequilibrium is not uncommon in consangious populations such as where the study is being conducted. [17]

### Multiloci genotype association with risk of disease severity across the tuberculosis disease spectrum

We have previously shown that IFNγ/IL10 ratio is a critical determinant of disease severity [16]. Since both cytokines (IFNγ, IL10) showed distinct associations with different sites and severity of TB, we also assessed if genotype combinations for these two cytokines may contribute to increased risk of TB with respect to site or severity. There were nine possible combinations. Only significant results are shown in Table 5. As expected from previous analyses (Table 3) there was a clear association of IFNγ *TT* ^hi^ with pulmonary disease while IFNγ *AA^lo^* was clearly associated with increased risk of PAD and DTB. The effect of IL10 genotype combination was much more evident with disease site rather than disease severity. IL10 *GG* ^lo^ in combination with IFNγ *AA^lo^* was associated with the highest risk for DTB (OR = 3.59; *p* = 0.045). The results were similar when cases were compared with TST+ controls.

## Discussion

Although the effect of cytokine gene polymorphisms (SNPs) on tuberculosis disease sites or disease severity have been reported in different populations, our results for the first time show differential association of cytokine genotype combinations with either pulmonary or extra-pulmonary disease. We also report that in the lung compartment the high responder IL10 −1082 *AA* ^hi^ may play a critical role in limiting tissue damage in PTB. Because of careful characterization of disease in each of the group, we are able to show significant differences between groups and among groups. In addition, differences seen in both cytokines SNPs can be reconciled with their biological function. We feel that this is a very important issue despite small numbers in the stratified groups. When pulmonary patients are not stratified the sample size is sufficient for power of statistics. We have therefore given results for both pooled and stratified groups. There are very few studies, which relate genotype to phenotype. This is the strength of the current study.

There are considerable variations in allelic frequencies of cytokine gene polymorphisms in different populations [18] and therefore it is not surprising that genetic polymorphisms associated with tuberculosis have yielded conflicting results in different ethnic groups [19]. IFNγ polymorphism (+874 *T* ^hi^→*A* ^lo^) is the most studied polymorphism in terms of association with tuberculosis disease sites and severity. However, the reports are conflicting in that *A* allele is more common in patients with TB and *T* alleles, more common in controls in Italian [20], South African [21] Hong Kong Chinese [22]and Spanish populations [23]. In Turkey, one study reported no association [24], while a later study in the same population showed an association with the *A* allele [25]. In Croatia [26] an association with *A* allele was found only in microscopy and culture positive *vs.* negative TB patients. On the other hand, no association has been reported in Caucasians in Houston, Texas [27] and South Indian populations [28]. There is only one study in Colombia [29] where an association of IFNγ +874 *T* allele with the more localized pleural disease has been observed. Our results are in agreement with the latter study. We show that the effect of IFNγ *T* allele in TB affected patients is restricted to pulmonary patients with minimal/ moderate disease increasing the risk by 2–3 folds (Table 4). Both our study and the Colombian study show a much more marked association with the more protective form of tuberculosis. Some of the differences could be due to the influence of other genes linked to tuberculosis disease susceptibility [10], [28], [30]–[32]. As shown in our study, the presence of high responder IL10 −1082 *AA* ^hi^ may limit lung tissue damage when associated with IFNγ *TT* ^hi^. Turner has reported *AA* to be a low producer phenotype in response to mitogenic (ConA) stimulus [33]. Mycobacterial antigens are potent stimulators of macrophages [34] while Con A is primarily a T cell stimulator [33]. Therefore, it is possible that different stimuli may result in differential transcription of the same gene. For the current study we considered mycobacterial antigens to be the more appropriate stimulus.

A similar discrepancy is observed with IL10 (−1082 *G* ^lo^→*A* ^hi^) where no associations were reported in Gambian [35], Korean [36] and Spanish [23] populations. An association of *A* allele was observed in Italian (Sicilian) [37] population, and *GA* heterozygosity was associated with pulmonary TB in Cambodia [38]. Our results indicate that the homozygous *GG* ^lo^ increases the risk of DTB when it is associated with IFNγ +874 *AA* ^lo^ phenotype (OR = 3.59; p = 0.045). This is consistent with the low levels of mycobacterial stimulated IFNγ and IL10 from DTB [39]. We have observed an overrepresentation of *GA* heterozygous genotype in our study groups and may increase the risk of PMN when associated with IFNγ *AA* ^lo^ (Table 5). The frequency of *GA* heterozygosity is highly variable in different populations ranging from 82.5% in Iranian population to 5% in Singapore Chinese [18]. Our results (70.2%) are closer to the Iranian population frequency. We further confirmed PCR amplified product by sequencing which also gave the expected SNP sequences for both IFNγ and IL10 genotypes. Therefore, it is difficult to attribute the inflation in *GA* heterozygosity to artifacts in genotyping methodology as most studies have reported widely differing frequencies using ARMS PCR [18]. It is not unexpected to find linkage disequilibrium in consangious population such as the setting in which this study was conducted [17]. Further population-based studies are needed to address this issue.

Nevertheless, a consensus seems to be emerging in that the combined effect of several cytokine SNPs may play a more crucial role in disease severity [29]. These results also substantiate our earlier report [16] that IFNγ/IL10 ratio may be the critical determinant of clinical severity in both pulmonary and extra-pulmonary tuberculosis. A meta analyses recently published reinforces the critical importance of IFNγ +874 T/A as a genetic marker for TB resistance [9], while IL10 indeed had some specific effect on TB determining the disease form and severity and not with susceptibility per se. Our results are consistent with these findings.

If the rationale for analyzing cytokine gene polymorphism is to understand the pathogenesis of human disease, to identify potential markers of susceptibility or disease severity, responder *vs.* non-responders in therapeutic and vaccine trials, and to design novel strategies for intervention in high-risk groups, then the diversity of genetic associations warrants that such analyses are carried out in indigenous population. Our results further highlight the importance of stratification of patients in relation to disease severity, which otherwise mask the significance of associations in combined groups.

## Materials and Methods

### Subject studied

Gene polymorphisms were analyzed in 376 donors (TB not affected, TBNA = 188 and TB affected, TBA = 188). Table 1 shows the breakdown of TB patients in terms of clinical severity: WHO guidelines for disease classification for non HIV related tuberculosis was adopted [16], [40] as follows. DTB (N = 20) had involvement of two or more sites with primary focus as meninges (N = 3), spinal (N = 1), intestinal (N = 11), splenic (N = 3) or miliary (N = 4) with or without lung involvement. PTB (N = 111) had involvement of lung parenchyma exclusively. LTB (N = 47) included TB restricted to one site without lung involvement (lymph nodes = 29, peripheral joints = 9, pleural = 8 and endobronchial = 1). Patients were included in the study if they were positive by one or more of the following criteria: microscopy, culture, histology, imaging (chest×rays for pulmonary patients and miliary involvement, CT scans for abdominal and skeletal and splenic involvement, MRI for meninges, or definitive clinical response (Table 2).

**Table 2:** Diagnostic modality used for confirmation of tuberculosis.

Disease* category	N	% (TST≥10 mm)	Microscopy	Culture	Histology	¶Imaging	Response to treatment
TBNA	188	71	-	-	-	-	-
Pulmonary	111	62	47	24	0	38	2
Disseminated	20	35	1	3	2	12	2
Localized	47	43	1	10	13	10	13

Note: Primary diagnostic modality used diagnosis of tuberculosis.

* Criteria for disease category given in material and methods.

¶ Imaging tests included chest×rays for pulmonary patients, CT scan and or MRI for disseminated disease.

**Table 3:** Genotype frequencies in healthy controls and different clinical forms of tuberculosis (TB).

Genotypes	TBNA (188)	TBA (188)	PTB (111)	PMN (19)	PMD (63)	PAD (29)	ETB (67)	DTB (20)	LTB (47)
**IFN-γ (+874)**									
**TT**	25 (13.3)	39 (20.74)	27 (24.32)	8 (42.11)	16 (25.4)	3 (10.34)	12 (17.91)	3 (15.00)	9 (19.15)
**TA**	87 (46.28)	83 (44.15)	47 (42.34)	6 (31.58)	28 (44.44)	13 (44.83)	30 (44.78)	8 (40.00)	22 (46.80)
**AA**	76 (40.43)	66 (35.11)	37 (33.33)	5 (26.32)	19 (30.16)	13 (44.83)	25 (37.31)	9 (45.00)	16 (34.04)
***χ^2^***		3.86	6.05	10.69	5.58	0.30	0.87	0.29	1.28
**Corrected ** ***p*** ** = **		0.08	**0.034**	**0.012**	**0.029**	0.588	0.433	0.859	0.278
**Corrected ** ***p*** ** = **	**TBNA TST+ (133)**	0.064	**0.007**	**0.029**	**0.008**	0.780	0.649	0.857	0.495
**IL-10 (−1082)**									
**GG**	20 (10.64)	27(14.36)	19 (17.12)	4 (21.05)	7 (11.1)	8 (27.59)	8 (11.94)	4 (20.00)	4 (8.51)
**GA**	136 (72.34)	132 (70.21)	71 (63.96)	11 (57.893)	46(73.02)	14 (48.25)	51 (76.12)	12 (60.00)	39 (82.98)
**AA**	32 (17.02)	29 (15.43)	21 (18.92)	4 (21.05)	10 (15.89)	7 (24.14)	8 (11.94)	4 (20.00)	4 (8.51)
***χ^2^***		1.25	3.09	2.20	0.05	8.41	0.98	1.85	2.52
**Corrected ** ***p*** ** = **		0.33	0.489	0.621	0.831	0.374	0.384	0.612	0.437
**Corrected ** ***p*** ** = **	**TBNA TST+ (133)**	0.435	0.767	0.945	0.867	0.341	0.330	0.582	0.357

Note: Patient stratification is given in Materials and Methods. N for each group given in brackets. Abbreviations used as in Table 1. Number (frequency) of genotypes is indicated. Pearson chi analysis was carried to determine the significance of differences.

All significant p values are indicated in bold. P approaching significance is given in italics. *p*<0.05 is considered significant.

**Table 4:** Differences in allele frequencies in healthy controls and tuberculosis patients

IFN-γ (+874 T^hi^→A^lo^)	N	chi-value	corrected *p* =	OR	95% CI (lower)	95% CI (upper)
**TBNA**	**188**					
**TBA**	**188**	3.20	0.074	1.31	0.79	1.75
**PTB**	**111**	4.78	**0.029**	1.46	1.04	2.04
**PMN**	**19**	6.72	**0.01**	2.4	1.22	4.72
**PMD**	**63**	4.95	**0.026**	1.59	1.05	2.38
**PAD**	**29**	0.29	0.587	0.85	0.47	1.53
**ETB**	**67**	0.46	0.50	1.15	0.77	1.72
**DTB**	**20**	0.032	0.858	0.94	0.47	1.86
**LTB**	**47**	1.12	0.274	1.29	0.82	2.05
**IL-10 (−1082 G^lo^**→**A^hi^)**						
**TBNA**	**188**					
**TBA**	**188**	0.53	0.48	1.11	0.83	1.48
**PTB**	**111**	0.29	0.58	1.01	0.79	1.54
**PMN**	**19**	0.14	0.708	1.14	0.58	2.22
**PMD**	**63**	0.025	0.875	1.03	0.69	1.55
**PAD**	**29**	0.49	0.486	1.22	0.7	2.12
**ETB**	**67**	0.036	0.85	1.04	0.7	1.54
**DTB**	**20**	0.15	0.701	1.14	0.59	2.18
**LTB**	**47**	0.307	0.58	1.14	0.72	1.79

Note: Patient stratification is given in material and methods. N for each group is given in brackets. Abbreviations used as in Table 1.

**Table 5:** Genotype combination in relation to disease severity.

IFNγ/IL10 genotypes	TBNA	PMN	PMD	PAD	DTB	LTB
N	188	19	63	29	20	47
**TT/GG %**	2.13	10.53	1.59	6.9	0	4.2
chi^2^		6.66				
**corrected ** ***p*** ** = **		**0.01**				
*OR*		*6.06*				
*(95% CI)*		*(1.30–28.07)*				
**TT/AA %**	3.19	10.53	6.35	3.45	5	2.13
chi^2^		4.92				
**corrected ** ***p*** ** = **		**0.027**				
*OR*		*3.99*				
*(95% CI)*		*(1.08–14.8)*				
**TT/GA%**	7.98	21.05	17.46	0	10	12.8
chi^2^		6.82	4.4			
**corrected ** ***p*** ** = **		**0.009**	**0.036**			
OR		*3.06*	*2.50*			
(95% CI)		*(1.08–7.28)*	*(1.04–6.1)*			
**TA/GG %**	5.85	0	4.76	6.9	10	2.13
chi^2^						
**corrected ** ***p*** ** = **						
*OR (95% CI)*						
**TA/AA %**	5.32	10.53	6.35	6.9	0	4.2
chi^2^						
**corrected ** ***p*** ** = **						
*OR (95% CI)*						
**TA/GA %**	35.11	21.05	33.33	31.03	30	40.4
chi2 (*p*-value)		4.86				
**corrected ** ***p*** ** = **		**0.028**				
*OR*		*0.40*				
*(95% CI)*		*(0.26–0.93)*				
**AA/GG %**	2.66	10.53	4.76	13.79	10	2.13
chi^2^		4.92		7.78	4.03	
**corrected ** ***p*** ** = **		**0.027**		**0.005**	**0.045**	
*OR*		*3.99*		*5.26*	*3.59*	
*(95% CI)*		*(1.08–14.79)*		*(1.46–18.9)*	*(0.96–13.47)*	
**AA/AA %**	8.51	0	3.17	13.79	15	2.13
chi^2^						4.71
**corrected ** ***p*** ** = **						**0.03**
*OR*						*0.21*
*(95% CI)*						*(0.04–0.98)*
**AA/GA %**	39.26	15.79	22.2	17.24	20	29.7
chi^2^		4.85		4.06		
**corrected ** ***p*** ** = **		**0.028**		**0.044**		
*OR*		*0.45*		*0.50*		
*(95% CI)*		*(0.24–0.93)*		*(0.25–0.99)*		

The control group consisted of healthy donors (TBNA = 188) with no signs, symptoms or history of previous tuberculosis. Tuberculin skin tests (TST) positivity was assessed by administering five tuberculin units intracutaneously on the volar surface of the right arm. An induration of ≥10 mm was used as a cut for positive responses (TST+) which is considered to be indicative of latent infection. The Aga Khan University Ethical Review Committee (ERC) approved the project. Written consent was obtained, for each participant or his or her guardians in case of minors after explaining the purpose of the study.

### DNA Extraction

Five ml blood were collected in ACD (VWR Scientific, West Chester, PA, USA) tubes and kept frozen until use. Genomic DNA was extracted from frozen whole blood using Promega Wizard Genomic DNA Purification Kit (Promega Corporation Madison, WI, USA) according to the manufacturer's instructions. After extraction, DNA was quantified by spectrophotometery, checked for purity and stored at −35°C until further analyses.

### Molecular analysis

IFNγ and IL10 genotype analyses were carried out using amplification refractory mutation system-PCR (ARMS-PCR) [41]. Primers used for the detection of SNPs were purchased from MWG-Biotech AG, (Ebersberg, Germany). Human growth hormone and β actin were used as internal controls to check the accuracy of PCR reactions. Amplified products were monitored by electrophoresis on agarose gel containing 10 mg/ml ethidium bromide. Product bands were visualized on a transiluminator and polaroid pictures were taken for interpretation.

Sequencing methodology was used as a second confirmatory method on a subset of samples to verify that correct alleles were being identified by ARMS-PCR. The primers used for sequencing analyses were IFNγ Forward (5′-TAT GAT TCT GGC TAA GGA-3′), IFNγ Reverse (5′-CCC CAA TGG TAC AGG TTT CT-3′) and IL-10 Forward (5′-TGT GGA AGG GGA AGG TG-3′), IL-10 Reverse (5′-TAA AAG ATG GGG TGG AAG AA-3′). These primers were designed using software Lasergene version 7.0 (DNAstar, Madison, WI, USA) and amplify a part of the gene that covers IFNγ (+874T→A) or IL10 (−1082G→A) SNPs to yield products size of 318 and 329 bp respectively. PCR products were purified with the QIAquick PCR purification kit (Qiagen, Hilden, Germany) according to the manufacturer's instructions. Purified products were then sent to Macrogen for sequencing (Macrogen Inc, Seoul, Korea) with both forward and reverse primers. Sequencing results were analyzed by BLAST search of the GenBank database and EMBOSS pairwise alignment of the EMBL-EBI database. There was complete concordance between PCR based and sequence based analyses for homozygous as well as heterozygous genotypes (+874, *TT* = 13; *AA* = 9; −1082, *GG* = 6; *GA* = 17).

### Whole blood stimulation assays

Stimulated whole blood (WB) culture assay for assessing cytokine profiles have been described in detail previously [34]. Briefly, heparinized blood was diluted 1/11 with sterile RPMI 1640 tissue culture medium containing 100 units/ml of penicillin/100 µg/ml streptomycin and 2 mM L-glutamine (Sigma Chemical Co., St Louis, Mo). Diluted WB (900 µl/ well) were stimulated with MTB culture filtrate proteins [5 µg/m] in a 24-well tissue culture plates (Flow Laboratories, Irvine, Scotland) within 2 hours of collection. Supernatants were collected from the wells at varying intervals and stored as 4×200 µl aliquots at −35°C.

### Cytokine assessment

Cytokine (IFNγ and IL10) in supernatants were assessed using pairs of monoclonal antibodies as described in detail previously [39]. Dose response curves were set up in each individual plate. Supernatants were serially diluted and optical density readings in the linear range of the dose response curve were used for calculating the concentrations. The final concentrations (pg/ml) were obtained after multiplying the values by dilutions at which the OD was read. The sensitivity and range of cytokine detection was 7.5–1000 pg/ml and was comparable to that reported by the manufacturer.

### Statistical Analysis

Allelic and genotypic frequencies and multi loci analysis were compared for all groups together and for individual patient and control groups. Computer software SPSS version 16.0 and Epi Info 2000 applications were used to carry out statistical analyses. Frequencies were compared between groups by Pearson chi-squared tests or Fisher's exact tests, when analyzing allelic frequencies lower than five to determine statistical significance differences between groups. Odds ratios (OR) with respective confidence intervals (95% CI) for disease susceptibly were also calculated. Linear-by-linear test were used to determine the significance (corrected p values) of genotypes between TB groups and healthy controls. Multiple logistic regression analysis was applied, to determine the effect of age and sex with genotypes. Values of *p*<0.05 were considered significant for both Pearson and linear-by-linear χ2 test. Hardy-Weinberg proportions were determined by applying the equation (p^2^+2pq+q^2^).
